# Elevated levels of MMP12 sourced from macrophages are associated with poor prognosis in urothelial bladder cancer

**DOI:** 10.1186/s12885-023-11100-0

**Published:** 2023-06-30

**Authors:** Iliana K. Kerzeli, Alexandros Kostakis, Polat Türker, Per-Uno Malmström, Tammer Hemdan, Artur Mezheyeuski, Douglas G. Ward, Richard T. Bryan, Ulrika Segersten, Martin Lord, Sara M. Mangsbo

**Affiliations:** 1 Department of Pharmacy, Science for Life Laboratory, Uppsala University, Uppsala, Sweden; 2 Department of Surgical Sciences, Uppsala University, Uppsala, Sweden; 3 Department of Immunology, Genetics and Pathology, Uppsala University, Uppsala, Sweden; 4 Vall d’Hebron Institute of Oncology, Barcelona, Spain; 5 Bladder Cancer Research Centre, Institute of Cancer & Genomic Sciences, College of Medical & Dental Sciences, University of Birmingham, Birmingham, UK

**Keywords:** Biomarkers, Matrix metalloproteinase 12 (MMP12), Urothelial bladder cancer, Single-cell transcriptomics, Proteomics, Proximity Extension Assay (PEA), Macrophages

## Abstract

**Background:**

Urothelial bladder cancer is most frequently diagnosed at the non-muscle-invasive stage (NMIBC). However, recurrences and interventions for intermediate and high-risk NMIBC patients impact the quality of life. Biomarkers for patient stratification could help to avoid unnecessary interventions whilst indicating aggressive measures when required.

**Methods:**

In this study, immuno-oncology focused, multiplexed proximity extension assays were utilised to analyse plasma (*n* = 90) and urine (*n* = 40) samples from 90 newly-diagnosed and treatment-naïve bladder cancer patients. Public single-cell RNA-sequencing and microarray data from patient tumour tissues and murine OH-BBN-induced urothelial carcinomas were also explored to further corroborate the proteomic findings.

**Results:**

Plasma from muscle-invasive, urothelial bladder cancer patients displayed higher levels of MMP7 (*p* = 0.028) and CCL23 (*p* = 0.03) compared to NMIBC patients, whereas urine displayed higher levels of CD27 (*p* = 0.044) and CD40 (*p* = 0.04) in the NMIBC group by two-sided Wilcoxon rank-sum tests. Random forest survival and multivariable regression analyses identified increased MMP12 plasma levels as an independent marker (*p* < 0.001) associated with shorter overall survival (HR = 1.8, *p* < 0.001, 95% CI:1.3–2.5); this finding was validated in an independent patient OLINK cohort, but could not be established using a transcriptomic microarray dataset. Single-cell transcriptomics analyses indicated tumour-infiltrating macrophages as a putative source of MMP12.

**Conclusions:**

The measurable levels of tumour-localised, immune-cell-derived MMP12 in blood suggest MMP12 as an important biomarker that could complement histopathology-based risk stratification.

As MMP12 stems from infiltrating immune cells rather than the tumor cells themselves, analyses performed on tissue biopsy material risk a biased selection of biomarkers produced by the tumour, while ignoring the surrounding microenvironment.

**Supplementary Information:**

The online version contains supplementary material available at 10.1186/s12885-023-11100-0.

## Background

Urothelial bladder cancer (UBC) is the 10^th^ most prevalent cancer worldwide and the 4^th^ among men [1]. Long-term surveillance of patients at risk of recurrence or progression is costly for healthcare providers and burdensome for patients, necessitating repeated cystoscopies and imaging. However, existing classifications, which depend solely on clinicopathological variables [2, 3], are insufficient for the precise prediction of treatment responses or disease trajectory. Consequently, there is an urgent and unmet need to identify accurate local and systemic predictive and/or prognostic biomarkers. Proximity Extension Assays (PEAs) are sensitive tools for biomarker discovery, but are yet to be explored in-depth in liquid biopsies from UBC patients.

UBC is associated with carcinogen exposure, often resulting in tumours characterised by high mutational load [4, 5]; such features may lead to immunogenicity and an immunotherapy-responsive disease by increasing the likelihood of HLA-presentable tumour neoantigens that are able to initiate an immune response [6–8]. High numbers of macrophages [9–11], neutrophils [12], and myeloid-derived suppressor cells [13, 14] in UBC are further associated with poor survival and limited treatment responses.

This study aimed to investigate whether a sensitive protein detection method such as PEA technology could aid in prognostic biomarker discovery in liquid biopsies by exploring the systemic and local proteomic contexture of urothelial cancer in relation to immune- and tumour-related processes. To achieve this, we utilised an immuno-oncology-focused multiplexed PEA panel to analyse plasma and urine samples from a Swedish UBC patient cohort. Findings were validated using PEA data from an independent UK UBC cohort [15], and publicly-available RNA-sequencing data were interrogated to identify gene expression changes and the cellular sources of the prognostic biomarkers.

## Methods

**Fig. 1:**
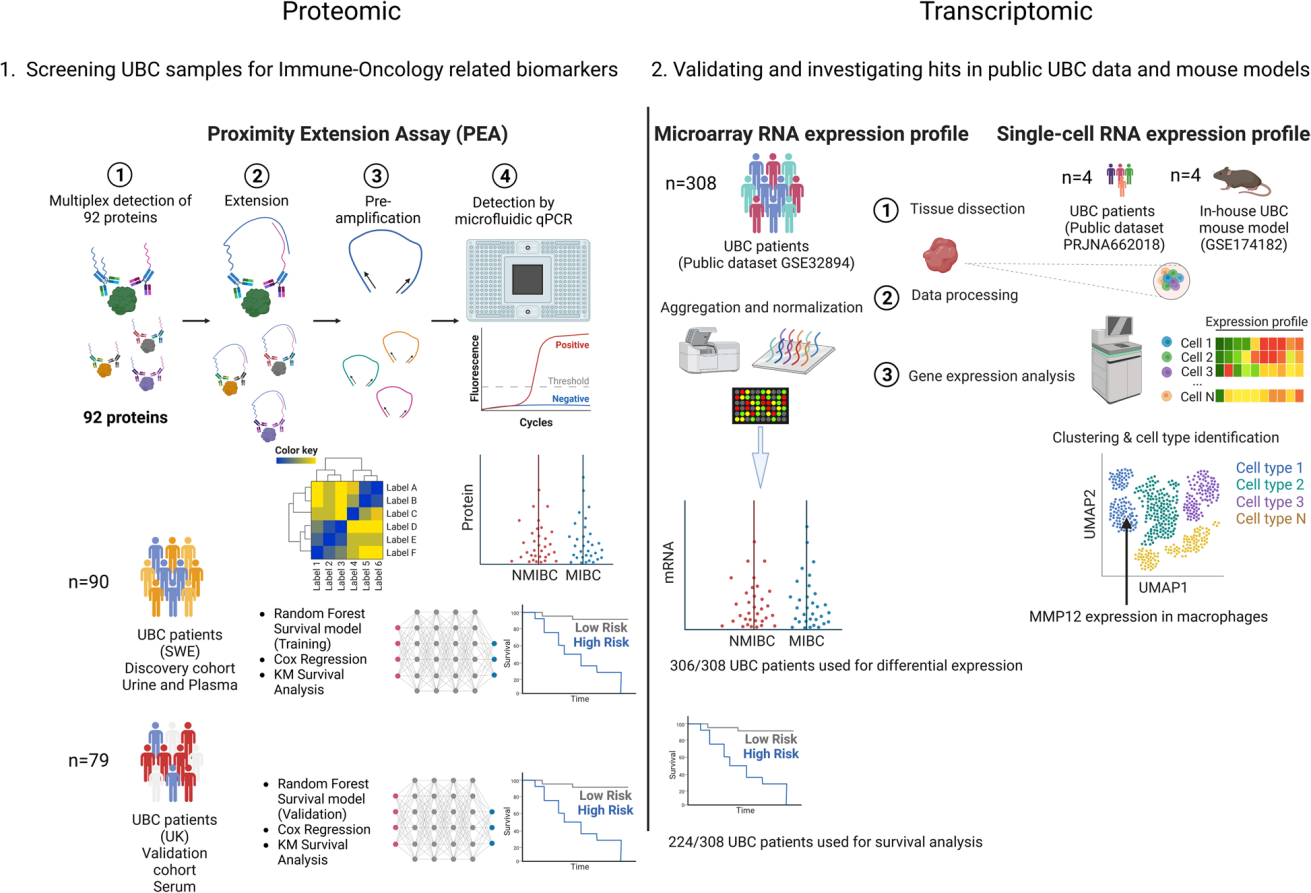
A graphical overview of the methods, cohorts and analysis workflows presented in this study. An initial 1) screen of immune-oncology-related proteins in plasma/serum was performed using OLINK, which was followed by 2) the use of transcriptomics to further investigate and validate the proteomic findings. Patient numbers are referred to as *n* with public datasets presented next to each transcriptomic assay

A graphical illustration of the overall analysis workflow and aims was created with BioRender.com and is presented in Fig. 1.

### Study populations

Patients included in the discovery cohort were diagnosed with UBC between 1986 and 1996 at the University Academic Hospital of Uppsala, Sweden. The study was performed in accordance with the Declaration of Helsinki and approved by the regional ethical review board of Uppsala (ID 2015/143/1). Plasma samples (collected in heparin tubes) from 90 of these patients and paired urine samples (morning urine) from 40 (33/40 passed QC) were stored at -80 °C and were not thawed until their analysis with PEA as described below. Reference pathology was performed by an experienced pathologist according to the 2004 WHO Classification of Bladder Tumours [16]. Clinical and histopathological characteristics are presented in Table 1. The clinical follow-up of the patients was conducted in accordance with the European and National Guidelines with regular cystoscopies and transurethral resection of the tumours when recurrences had been detected*.* For validation, we utilised PEA data derived from the pre-treatment serum samples of 79 UBC patients recruited to the West Midlands’ Bladder Cancer Prognosis Programme (BCPP), UK (ethical approval: 06/MRE04/65) [15, 17].

**Table 1:** Clinical characteristics and demographic data of the main screening urothelial bladder cancer cohort were used in this study. Within the Recurrences group, “Few” is defined as less than three recurrent tumours within 18 months, whereas “Frequent” is defined as three or more recurrences within the same period [18]

	Plasma *n* = 90	Urine *n* = 33
Sex		
Female	23 (26%)	11 (33%)
Male	67 (74%)	22 (67%)
Age	72 (64, 77)	72 (64, 78)
Stage Grade		
Ta Low grade	21 (23%)	7 (21%)
Ta High grade	10 (11%)	3 (9%)
T1 Low grade	3 (3%)	1 (3%)
T1 High grade	27 (30%)	10 (30%)
T2, T3, T4 High grade	29 (32%)	12 (36%)
Invasiveness status		
NMIBC	61 (68%)	21 (64%)
MIBC	29 (32%)	12 (36%)
Recurrences		
No	26 (29%)	11 (33%)
Few	37 (41%)	10 (30%)
Frequent	16 (18%)	6 (18%)
Unknown	11 (12%)	6 (18%)
Progressed to MIBC		
No	65 (72%)	24 (73%)
Yes	18 (20%)	7 (21%)
Unknown	7 (8%)	2 (6%)

n (%); Median (IQR)

### Multiplex proximity extension assay

Levels of 92 immuno-oncology-related proteins (OLINK Immune Oncology panel I) in urine and plasma were quantified by PEA [19] (Proseek platform, OLINK) at the Clinical Biomarker facility (Science for Life Laboratory, Uppsala, Sweden). Briefly, 92 proteins were detected in 1 μl of sample in a multiplex assay utilising concomitant binding of matched oligonucleotide-conjugated antibody pairs. When in close proximity, the corresponding antibody oligonucleotide pairs hybridize, resulting in a unique DNA template. Following a polymerase amplification reaction, the sequences are quantified by microfluidic qPCR and presented as Normalised Protein eXpression (NPX), an arbitrary unit based on log_2_-transformed Ct values (http://www.olink.com).

### Microarray gene expression analysis

To complement the small cohort used for the OLINK assay and to examine whether the observed differences could be noted in a larger cohort, made up of tissue biopsies, microarray data for the tumours from 308 UBC patients (in the form of non-normalised, raw intensity values along with their detection *p*-values) were retrieved from the gene expression omnibus (GEO) repository (GSE32894); clinical information regarding the patients and their samples was obtained from the associated publication [20]. Two of the samples were excluded from further analysis because the stage was not reported. Quantile normalisation, probe filtering and batch effect correction were performed according to the methods supplied by the original authors. Differential expression analysis was conducted in an R 4.1 environment (limma, v.3.48.0 [21], R). A linear model comprising a design matrix without intercept for stage of each sample, as well as a contrast matrix stating the comparisons to be made were fitted to the data to calculate fold changes, with positive values indicating higher expression in muscle invasive bladder cancer (MIBC) and negative values reflecting higher expression in non-muscle invasive bladder cancer (NMIBC). Patients with available survival data (*n* = 224) were selected for subsequent survival analyses (survival 3.2.7, R), and were split into highly- or lowly-expressing for each queried protein with cutoffs determined by maximally selected rank statistics (maxstat v.0.7.25, R).

### Single-cell RNA-sequencing data analysis

Analysis of single-cell RNA-sequencing data from a murine UBC model and human UBC patients were explored with the goal of identifying the putative source of the mRNA transcripts coding for selected proteins from the OLINK assay. Two sets of single-cell RNA-sequencing data were downloaded and processed: unique molecular identifier (UMI) count matrices originating from bladders of a urothelial cancer mouse model [22] (GSE174182) and raw sequence reads derived from patient tumour material from the European Nucleotide Archive under the accession code PRJNA662018 [23]. To generate count matrices for patient samples, the raw sequences were aligned to the GRCh38 reference genome through CellRanger 2.2.0. Downstream processing followed the Seurat v4.0 pipeline [24]. Murine cells were filtered to retain higher quality cells (> 200 & < 8000 uniquely identified genes, < 25% of reads mapped to mitochondrial genes), whereas the filtering thresholds for the human samples remained identical to the original publication (< 6000 unique genes, > 1000 UMI counts per cell and < 10% reads stemming from mitochondrial DNA). SCTransform [25] was employed to normalise and cluster the murine-derived cells, with clusters annotated based on their top differentially expressed genes. As for the human-derived cells, an additional integration step was performed prior to clustering due to the highly patient-specific clusters observed.

### Statistical analyses

For group comparisons, a two-sided Wilcoxon rank-sum test was used with adjustment for multiple testing by the Benjamini–Hochberg procedure (PEA data), or Bonferroni (mRNA data). Due to the internal normalisation procedures applied for the quantification of each protein, NPX values cannot be compared between proteins. Instead, the strength of association between protein markers was interrogated by Spearman rank correlation within or between respective sample types (serum and urine). Receiver operating characteristic (ROC) curves were constructed from logistic regression. Proteins important for overall survival (OS) were retrieved from a random survival forest model (RSF) [26]. The ranking of proteins was based on their importance in classifying the data in the model using their variable importance (VIMP) measurements. The assembly model used herein was built on the time-to-event data of the cohort and used the intersection of the 63 markers shared between the OLINK Immuno-Oncology panel I and the 422 OLINK markers investigated in the serum of UBC patients by Bryan et al. [15] (Supplementary Table 1). NPX values were scaled and trained on the Swedish cohort of 90 plasma samples, followed by validation against 79 serum samples from the UK cohort. The machine learning model was developed in the R milieu (RStudio Team 2019, Boston, USA) using the packages caret (v.6.0.86) and randomForestSRC (v.2.9.3). Maximally selected rank statistics (maxstat v.0.7.25, R) were used to dichotomize protein concentrations (high/low) in Kaplan–Meier log-rank test analyses. Multivariate overall survival estimates were calculated from Cox proportional hazard regression models comprising the covariates stage, grade, sex and age (survival 3.2.7, R).

## Results

### Large heterogeneity of proteomic profiles in urine and plasma of urothelial bladder cancer patients

**Fig. 2:**
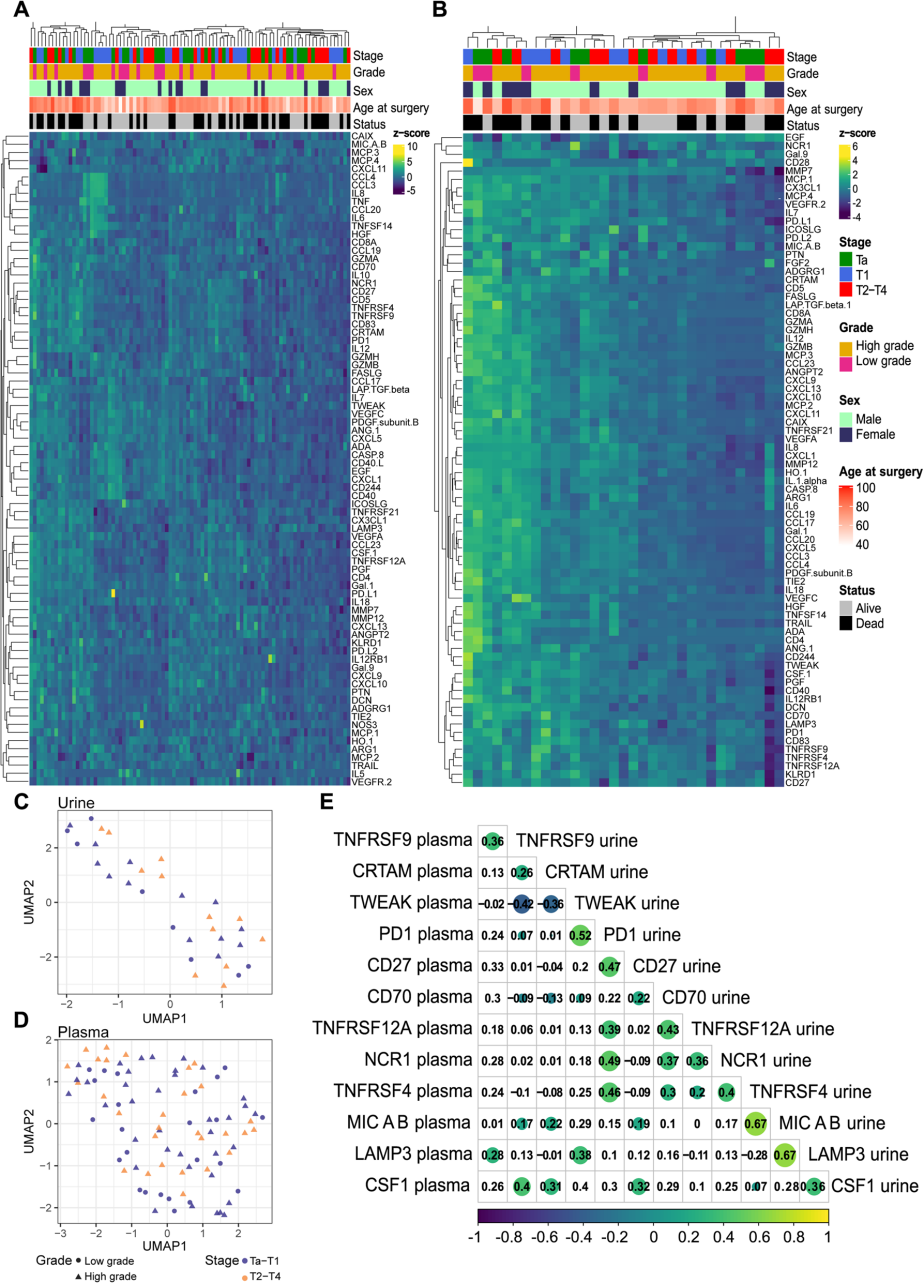
Immune-oncology protein marker profiles in plasma and urine of UBC patients. Unsupervised hierarchical clustering of patients based on (**A**) plasma and (**B**) urine protein NPX levels with corresponding clinical information presented above. Uniform manifold approximation and projection analysis of the overall protein marker levels in (**C**) plasma and (**D**) urine with Ta-T1 (NMIBC) and T2-T4 (MIBC) indicated in purple and orange, respectively. Circles represent low-grade and triangles high-grade tumours. **E** The correlation matrix for the 12 proteins significantly correlated between plasma and urine in paired samples (Spearman's rank-order correlation *p* < 0.05, *p-adj* < 0.05). Yellow represents a positive correlation and blue negative correlation with the size of the circle proportional to its R-value

Plasma and urine samples from a treatment-naïve UBC patient cohort were subjected to a highly sensitive targeted proteomic analysis (Immuno-oncology panel I, OLINK). Clinical and demographic data are presented in Table 1. From the initial screening of 92 protein panel markers, a subset was excluded from further analyses because NPX levels were below the limit of detection (LOD) in a large proportion of samples. Markers below LOD in plasma: IL-1α, IFN-γ, IL-33, IL-21, IL-35, CXCL12, FGF2, IL-2, IFN-β, IL-4, IL-13, CD28; and in urine: IL-5, IL-13, CXCL12, TNF, CD40L, IL-10, IL-4, IFN-β, NOS3, IL-21, IFN-γ, IL-2, IL-33, IL-35. Unsupervised hierarchical clustering and uniform manifold approximation and projection (UMAP) dimensional reduction analyses of the remaining proteins in plasma and urine revealed immuno-oncology profiles predominantly driven by differences among individuals as opposed to clinical variables such as stage, grade, sex or age **(**Fig. 2A-D**)**. The strength of the association between protein markers was investigated by Spearman rank correlation. The analysis revealed several proteins with significantly correlated urine and plasma concentrations from the 33 patients with paired samples. Following adjustment for multiple testing, 12 proteins involved in vital immune cell functions remained, with the strongest correlations observed for the surface-bound receptor/ligands LAMP3, MICA/B, PD-1 and CD27 (Fig. 2E).

### Stage-specific protein elevation in plasma and urine

**Fig. 3:**
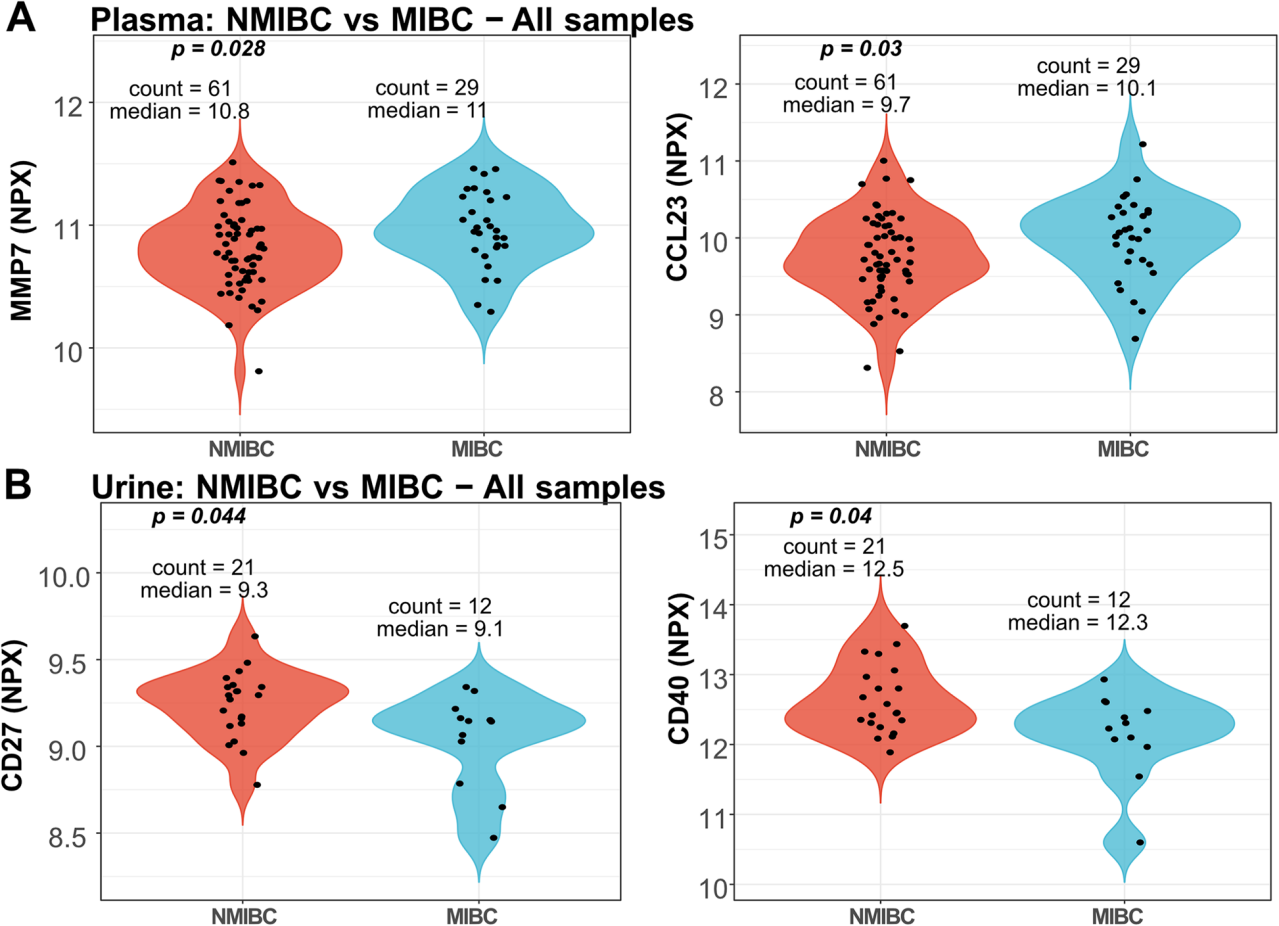
Differential protein expression between NMIBC and MIBC patients. **A** MMP7 and CCL23 NPX levels in plasma and (**B**) CD27 and CD40 levels in urine

NPX values of proteins were compared between NMIBC and MIBC patients. Systemic levels of matrix metalloproteinase 7 (MMP7) and C–C motif chemokine ligand 23 (CCL23) were significantly elevated in the plasma of MIBC patients compared to patients diagnosed with NMIBC (*p* = 0.028 and *p* = 0.03, respectively) (Fig. 3A); however, these levels were not statistically different in urine (*p* = 0.87 and *p* = 0.59, respectively). Instead, protein levels of the two TNF-receptor superfamily members, CD27 and CD40, were found to be present at significantly lower levels in the urine of MIBC patients compared to urine from NMIBC patients (*p* = 0.044 and *p* = 0.040, respectively) (Fig. 3B). To be noted, following correction for multiple testing, neither of the reported proteins reached significance (*q* < 0.05, FDR). When assessed as stage-specific classifiers in terms of sensitivity and specificity by calculating the area under the curve (AUC), the two urine markers CD27 (AUC = 0.71) and CD40 (AUC = 0.72) performed relatively well in combination (AUC = 0.75), whereas the plasma markers CCL23 (AUC = 0.64) and MMP7 (AUC = 0.64) alone, or in combination (AUC = 0.65) performed poorly (Supplementary Fig. 1).

### High MMP12 plasma levels are associated with poor overall survival outcome

**Fig. 4:**
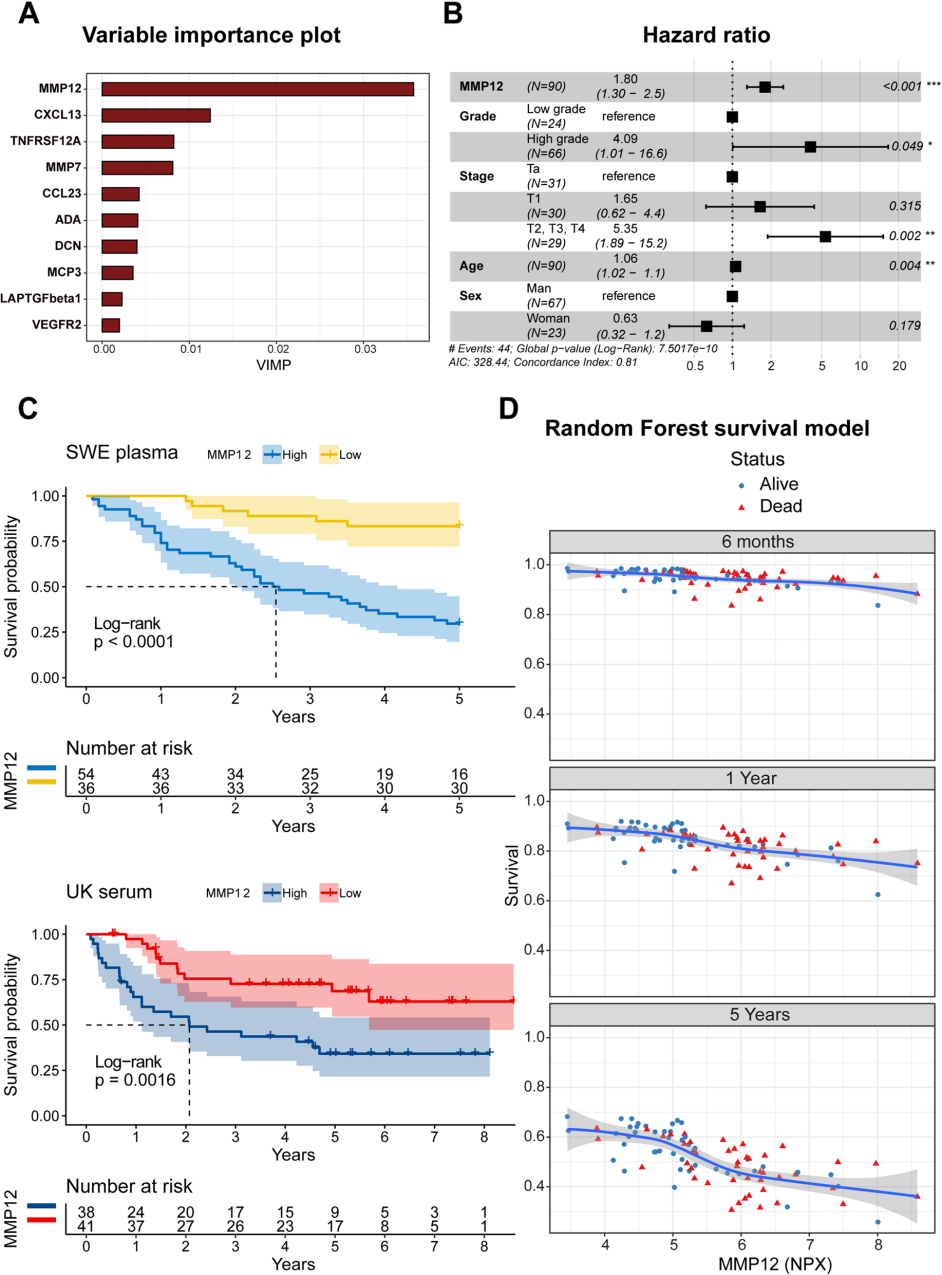
Proteins in plasma and serum are associated with overall survival. **A** The 10 most important plasma proteins for survival outcome were extracted from the plasma cohort RSF model (VIMP: Variable Importance). **B** Forest plot from the Swedish training data for MMP12, stage, grade, age and sex. High MMP12 levels are significantly associated with poor overall survival (hazard ratio > 1, *p* < 0.05). **C** Kaplan–Meier Survival Estimates curves from two urothelial cancer patient cohorts based on high or low MMP12 protein levels. Cut-off levels were calculated by maximally selected rank statistics. **D** RSF model predicted overall survival probability at different MMP12 NPX levels after 6 months, 1 year and 5 years

Compared to univariable or multivariate regression analysis with an individual protein, the RSF model included all proteins to predict survival status. To evaluate the accuracy of the model, data from a UK cohort [15] were used. The model included the 63 proteins shared between the Swedish and UK cohorts (Supplementary Table 1) and the respective patients’ overall survival status. Data from the Swedish cohort (90 plasma samples) were used as a discovery cohort to train the model with data provided by Bryan et al. [15] used as a validation cohort. Although not a perfect validation cohort (Swedish cohort = plasma, UK cohort = serum), the machine learning model presented an error rate of 32% given the different sample types. Here, MMP12 was ranked as the most important contributor to OS, as shown in the variable importance (VIMP) plot (Fig. 4A). The model’s main purpose was to identify prognostic biomarkers; thus, it did not include clinical variables. Instead, the top-ranked proteins were validated in a subsequent multivariate Cox-regression analysis comprising the covariates stage, grade, sex, and age (Supplementary Fig. 2, Supplementary Table 2A-B). When assessed as a continuous variable, MMP12 remained significantly associated with a poor outcome (HR = 1.8, CI:1.3–2.5, p < 0.001) in the training cohort (Fig. 4B) and in the validation cohort (HR = 2.20, CI:1.42–3.41, p < 0.001) (Supplementary Fig. 3, Supplementary Table 2C-D). The association between high levels of MMP12 and poor OS was maintained in the independent validation cohort (Fig. 4C). An interesting possibility with RSF models is the ability to study how MMP12 levels influence the predicted survival over time. Here, as a proof-of-concept, survival probability is presented at 6 months, 1 year and 5 years using the aforementioned model (63 markers, clinical information excluded) (Fig. 4D).

### Transcriptomic validation of MMP7 and MMP12 expression variation between NMIBC and MIBC

Due to the clinical importance of systemic levels of MMPs in this cohort, illustrated by stage specificity and survival differences, we sought to investigate their expression at the transcript level in datasets generated with complementary methods. We utilised a microarray-based dataset from a UBC patient cohort [20], as well as two single-cell RNA-sequencing datasets: a clinically relevant murine urothelial cancer model developed by our group [22] and a patient cohort comprising four non-invasive and four invasive tumours [23].

**Fig. 5:**
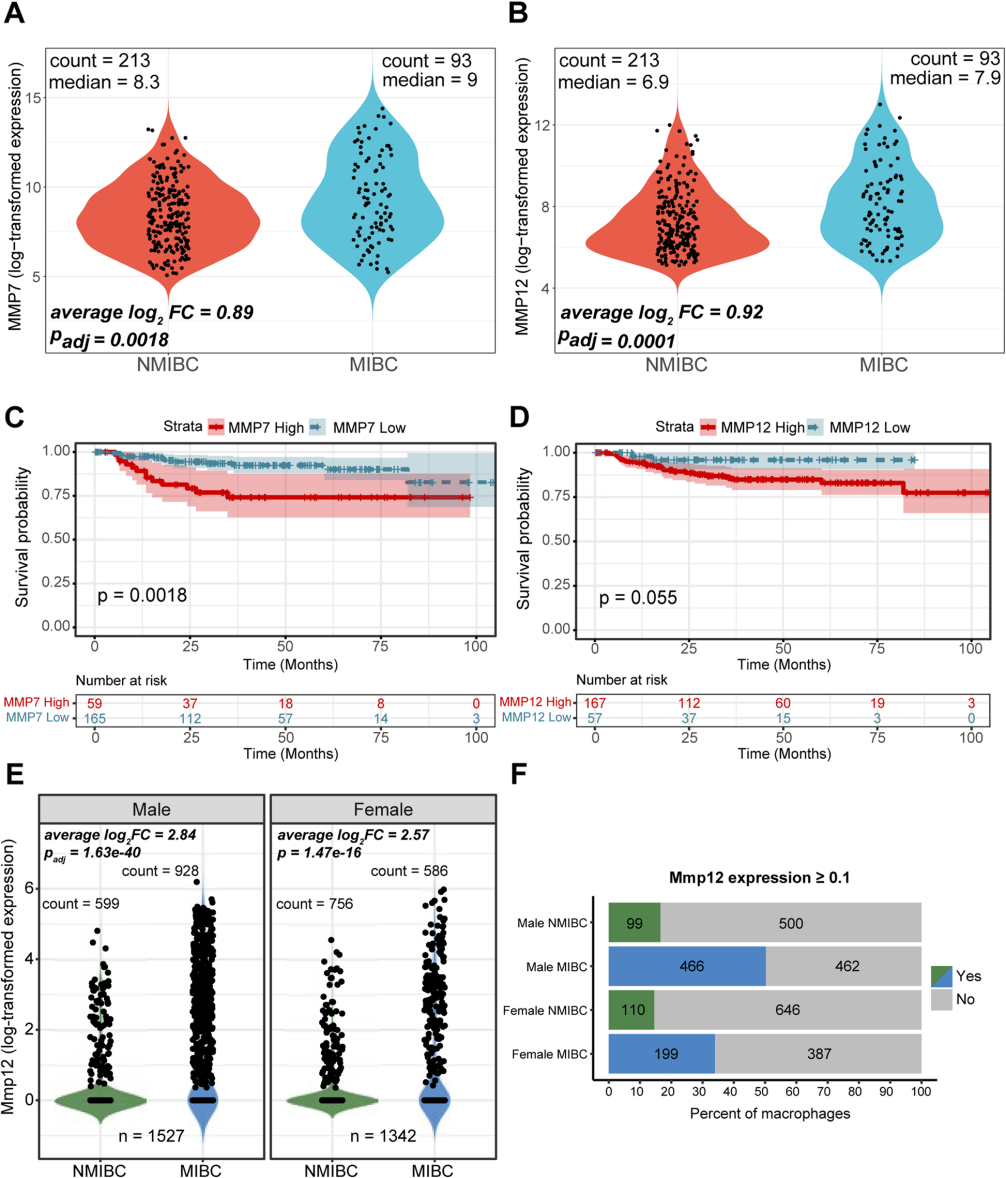
MMP7 and MMP12 expression in UBC patient biopsy tissues and an autochthonous murine urothelial cancer model. Normalised gene expression levels in tumour samples (*n* = 306) were analysed with microarray probes for *MMP7* (**A**) and *MMP12* (**B**) in relation to the patient’s stage. Kaplan–Meier plots for selected patients (*n* = 224) of the aforementioned cohort based on high or low mRNA levels of *MMP7* (**C**) and *MMP12* (**D**). *Mmp12* mRNA expression at the single-cell level from a murine urothelial cancer model is significantly higher at the MIBC stage, irrespective of sex (**E**). Bar plots illustrate the percentage and the total number of macrophages exhibiting non-zero expression of *Mmp12* (**F**) for the different stages and sexes. The average log_2_ fold changes and the BH-adjusted p-values were computed using limma for the microarray data, while the respective fold changes and Bonferroni-corrected p-values were calculated through a Wilcoxon rank-sum test provided by Seurat for the single-cell RNA-sequencing data

Analysis of the microarray gene expression data from 213 NMIBC and 93 MIBC patient tumour biopsies revealed that *MMP7* and *MMP12* mRNA expression levels were higher in MIBC. An approximately 1.85-fold increase for *MMP7* (*p-adj* = 0.0018) (Fig. 5A) and a 1.89-fold increase for *MMP12* (*p-adj* = 0.0001) (Fig. 5B) were observed for MIBC. Survival analyses in this cohort showed that patients with high expression of *MMP7* had statistically significant, poorer OS (*p* = 0.018) (Fig. 5C). The same trend was noticeable for patients with high expression of *MMP12*, however this comparison marginally failed to reach statistical significance (*p* = 0.055) (Fig. 5D). To be noted, the cohort examined was heavily skewed towards NMIBC patients.

Thus, we proceeded to study single-cell RNA data to fully capture the cellular source of the MMP findings. Subsequent examination of a public human urothelial cancer dataset (PRJNA662018) at the single-cell RNA level determined the source of these transcripts to be epithelial cells for *MMP7* and macrophages for *MMP12* transcripts (Supplementary Figs. 4 and 5). Notably, weak *MMP12* mRNA expression from epithelial cells was present in less than 10% of the total cells.

Single-cell transcriptomic analysis of murine tumours further corroborated the source of human tumour-derived *MMP12* expression to macrophages, as also murine *Mmp12* expression was mapped to macrophages **(**Supplementary Fig. 5). However, the expression of *Mmp7* was not adequately identified in the murine dataset. A total of 2869 cells were annotated as macrophages, 1355 of which originated from NMIBC and 1514 from MIBC murine samples (Fig. 5E). *Mmp12* expression was increased > sevenfold in male-derived macrophages (*p-adj* = 1.63^–40^) in MIBC compared to NMIBC and almost sixfold in female-derived macrophages (*p-adj* = 1.47^–16^) in MIBC compared to NMIBC (Fig. 5E). In addition to the elevated levels of *Mmp12* in MIBC, the percentages of *Mmp12-expressing* macrophages also increased as the disease progressed (Fig. 5F).

## Discussion

The majority of UBC patients require intensive, long-term surveillance which increases morbidity and healthcare-related costs. With the current standard-of-care treatments for NMIBC, 5-year recurrence-free survival ranges from 23 to 43%, while the 5-year progression-free survival ranges from 54 to 93% [27]. However, based solely on clinical and pathological characteristics, patient tumour recurrence and survival outcomes are difficult to predict. Although NMIBC and MIBC are molecularly complex and heterogeneous [28], recent developments in transcriptomic classification offer promise for better treatment stratification and personalised therapy [29, 30]. These approaches rely upon laboratory-based deep sequencing of tumour tissue, necessitating invasive biopsies, specialist public or commercial genomics laboratories, and a turnaround time of 5–10 days. Accurate protein-based prognostic biomarkers assessed in liquid biopsies could overcome some of these limitations and easily permit repeated assessments during the patient’s disease course.

In this study, we used an assay which combines high technical sensitivity and specificity with a high-throughput multiplex format, permitting the assessment of 92 protein markers of important immune- and tumour-related processes; the clinical samples were collected at the first diagnosis of UBC before therapeutic interventions. Nevertheless, stage-specific patient classification or clustering based on protein levels was not achieved – a result of the heterogeneity in proteomic patient profiles which did not follow stage- or grade-related patterns.

Released extracellular vesicles or shed immune-related receptors/ligands such as MICA/B, which was one of the 12 identified immune-related receptor/ligand proteins positively correlating between plasma and urine, may have a systemic impact on the immune response with an increased risk of developing immune evasion later in the disease trajectory in several cancer types [31].

Interestingly, we found that receptor-bound proteins were elevated in our liquid based analysis. The origin of elevated soluble CD40 in the urine of NMIBC compared to MIBC patients is likely to be antigen-presenting cells and its decreased levels at MIBC might be a biomarker of immune suppression in the local microenvironment, there are discrepancies as to whether increased expression of CD40 has beneficial or adverse effects on cancer prognosis and therapy responses [32, 33]. On the other hand, soluble CD27 is reduced in the serum of cancer patients compared to healthy individuals, while in the serum of cancer patients post immunotherapy, soluble CD27 correlates with improved survival probability [34].

MMP7 and CCL23, involved in tissue degradation and T cell recruitment signalling, were found to be significantly elevated in the plasma of MIBC patients compared to NMIBC patients. Significantly higher MMP7 levels were also observed at MIBC compared to NMIBC in the UK cohort [15], in gene expression data of an independent patient cohort [20], and in other studies [35]. In our study, analysis of tumour tissue gene expression indicated that *MMP7* was expressed at higher levels in tumours of MIBC patients compared to NMIBC.

Despite stage-specific differences, none of the aforementioned proteins reached a high UBC stage classification accuracy (AUC > 0.85). High levels of MMP7 have been reported to be associated with shorter overall survival in UBC [15, 36, 37]. However, neither MMP7 nor CCL23, CD27 or CD40 were significantly associated with overall survival when included as continuous variables in multivariable Cox-regression analyses (data not shown) in our dataset.

Instead, analysis of the data by application of an RSF model ranked increased plasma levels of MMP12 as the main driver for overall survival in the cohort, and as an independent prognostic biomarker associated with decreased overall survival of UBC patients following multivariable analysis comprising clinically relevant confounders, such as stage and grade. Due to the high inter-patient heterogeneity and collinearity between markers, a non-parametric and non-linear method, such as random forests, offers an alternative to classical approaches that are affected by many noise variables and high-order interactions between investigated variables [26]. Despite the differences in liquid biopsies (plasma and serum), sampling procedures, and the use of different multiplex panels, the association between high systemic MMP12 levels and poor survival were recapitulated in the UK cohort.

To evaluate if MMP7 and MMP12 can be identified as biomarkers when using clinical biopsies, we investigated their gene expression in 306 UBC patients [20]. Here, we found that MIBC had higher *MMP7* mRNA levels overall. Interestingly, compared to MMP12 protein levels, *MMP12* mRNA expression was significantly higher at the MIBC stage. However, when studying the impact of *MMP12* on survival in tissue biopsies analysed by transcriptomics, we could not corroborate the findings of the liquid biopsy data. This could be due to the bias introduced in the selection of the malignant cells retrieved for pathological scoring as well as the difference between transcriptomic versus proteomic analyses or the high number of NMIBC patients compared to late stage patients in the transcriptomics cohort. A biopsy for clinical diagnosis also focuses on sampling the most malignant tumor area, rather than the complete tumour microenvironment.

To explore the source of MMP7 and MMP12 in UBC, we analysed UBC patient tumour samples that underwent single-cell sequencing and identified epithelial cells as the source of *MMP7* and macrophages as the source of *MMP12* mRNA transcripts. Previous studies have shown that increased numbers of tumour-infiltrating macrophages correlate with advanced tumour stage and Bacillus Calmette–Guérin (BCG) treatment failure in UBC [38, 39]. The importance of sex in UBC in the context of immunomodulatory therapies is still not fully explored [40], additionally, macrophages are usually expressed at a higher number and in a more active state in females compared to males [41], thus we included that variable in the single-cell RNA analysis for *MMP12*. However, we did not see any sex dependent difference in that aspect.

The family of MMPs is largely orthologous between humans and mice, sharing structure and function [42]. Utilising single-cell RNA-sequencing data of an autochthonous murine model of UBC, *Mmp12* mRNA expression was attributed to tumour-infiltrating macrophages. As observed in the human single-cell transcriptomic dataset, expression was also elevated in MIBC compared to NMIBC in male and female mice. However, *Mmp7* mRNA transcripts in the murine data set were only detected in a minor subset of urothelial cells and its expression was inadequate to warrant further inspection.

MMPs degrade the tissue matrix by extracellular proteolysis, thus facilitating tumour cell migration, invasion and metastasis. Moreover, MMPs are involved in several physiological and tumour-supporting cellular processes, including loss of cell adhesion, tumour angiogenesis, cell proliferation, epithelial-to-mesenchymal transition and apoptosis [43]. They have been extensively analysed in UBC, which has revealed potential roles for certain MMPs as diagnostic markers and prognostic factors at different stages of the disease course [44]. MMP12 is linked to reduced overall survival rates in numerous cancers [45–47]; in UBC, certain genetic polymorphisms of the gene have been shown to increase invasiveness [48], while higher mRNA expression of *MMP12* in tumour tissue has been reported to correlate with higher tumour grade [49].

## Conclusion

The data presented here, and validated across two independent OLINK patient cohorts, represent the first report of MMP12 as a potential independent prognostic biomarker for UBC survival. Further, the information about the lymph node metastasis status does not exist in the Swedish training cohort, which is a limitation of our study. Another limitation is that the Swedish cohort does not include sex and age-matched control samples collected and stored at the same timepoint.

Future studies should focus on validating these findings across defined patient subgroups using liquid versus tissue based analyses, investigating the detailed mechanistic role of MMP12 on tumour progression and immune response regulation, and assessing the possibilities for its inhibition as a route to improving long-term survival in UBC patients. Our proteomic finding and the non-tumour origin of MMP12 also highlights the necessity that further studies should implement a holistic approach (meaning that profiling must consider all cells present in the tumour microenvironment) to avoid the risk of a selection bias for biomarkers that are tumour shed. This could complete the picture on how immune and stroma cells play a role in tumour invasiveness.

## Supplementary Information

**Additional file 1:** **Supplementary Table 1.** Intersect of multiplex panel protein markers between the Swedish and the UK cohort.

**Additional file 2:** **Supplementary Table 2.** Univariate and multivariate Cox regression analyses of the VIMP proteins in the Swedish cohort and MMP12 in the UK cohort.

**Additional file 3: Supplementary Fig. 1.**

**Additional file 4: Supplementary Fig. 2.**

**Additional file 5: Supplementary Fig. 3**.

**Additional file 6: Supplementary Fig. 4.**

**Additional file 7: Supplementary Fig. 5.**

## Data Availability

OLINK data files are available from the corresponding author on reasonable request. Microarray data for the tumours from 308 UBC patients (in the form of non-normalised, raw intensity values along with their detection *p*-values) were retrieved from the GEO repository (GSE32894, https://www.ncbi.nlm.nih.gov/geo/query/acc.cgi?acc=GSE32894); clinical information regarding the patients and their samples was obtained from the associated publication [20]. Two sets of single-cell RNA-sequencing data were downloaded and processed: unique molecular identifier (UMI) count matrices originating from bladders of a urothelial cancer mouse model [22] (GSE174182, https://www.ncbi.nlm.nih.gov/geo/query/acc.cgi?acc=GSE174182) and raw sequence reads derived from patient tumour material from the European Nucleotide Archive under the accession code PRJNA662018 (https://www.ebi.ac.uk/ena/browser/view/PRJNA662018) [23]. All data generated or analysed during this study are included in this published article and its supplementary information files.

## Abbreviations

AUC: Area under curve
BCG: Bacillus Calmette–Guérin
BCPP: Bladder cancer prognosis programme
DAPI: 4′,6-Diamidino-2-phenylindole
GEO: Gene expression omnibus
LOD: Limit of detection
MIBC: Muscle invasive bladder cancer
MMP: Matrix metalloprotease
NMIBC: Non-muscle invasive bladder cancer
NPX: Normalised protein expression
OH-BBN: N-butyl-N-(4-hydroxybutyl)nitrosamine
OS: Overall survival
PANCK: Pan-cytokeratin
PEA: Proximity extension assay
ROC: Receiver operating characteristic
RSF: Random survival forest
TMA: Tissue microarray
UBC: Urothelial bladder cancer
UMAP: Uniform manifold and projection
UMI: Unique molecular identifier
VIMP: Variable importance
WHO: World health organisation
