# Supplementary figures and images for: Elevated levels of MMP12 sourced from macrophages are associated with poor prognosis in urothelial bladder cancer
Source: BMC Cancer. 2023 Jun 30;23:605. doi: 10.1186/s12885-023-11100-0 (PMC10311740; doi:10.1186/s12885-023-11100-0)

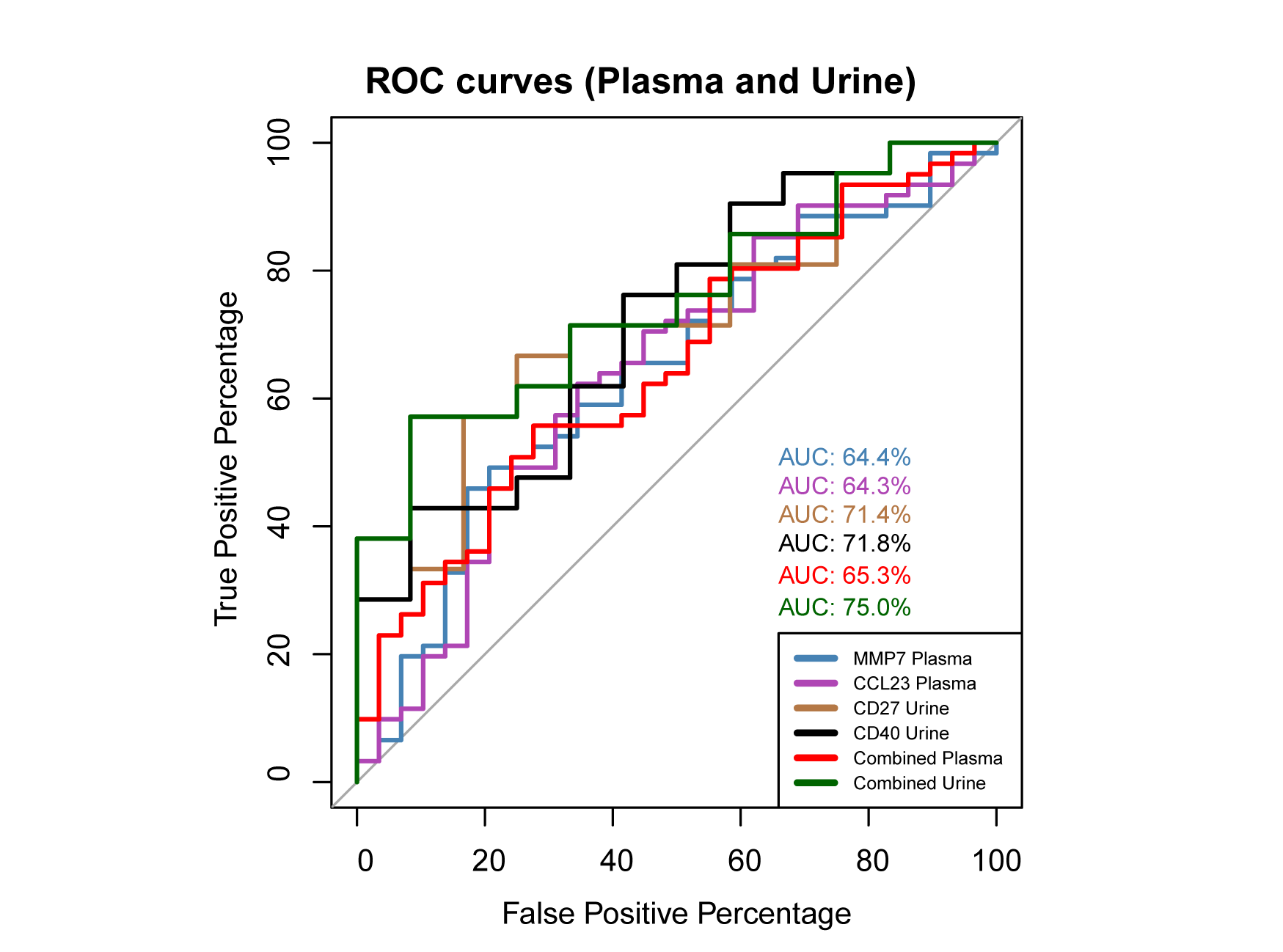

Supplement: Supplementary file 3 — Additional file 3: Supplementary Fig. 1. [file 12885_2023_11100_MOESM3_ESM.tif]

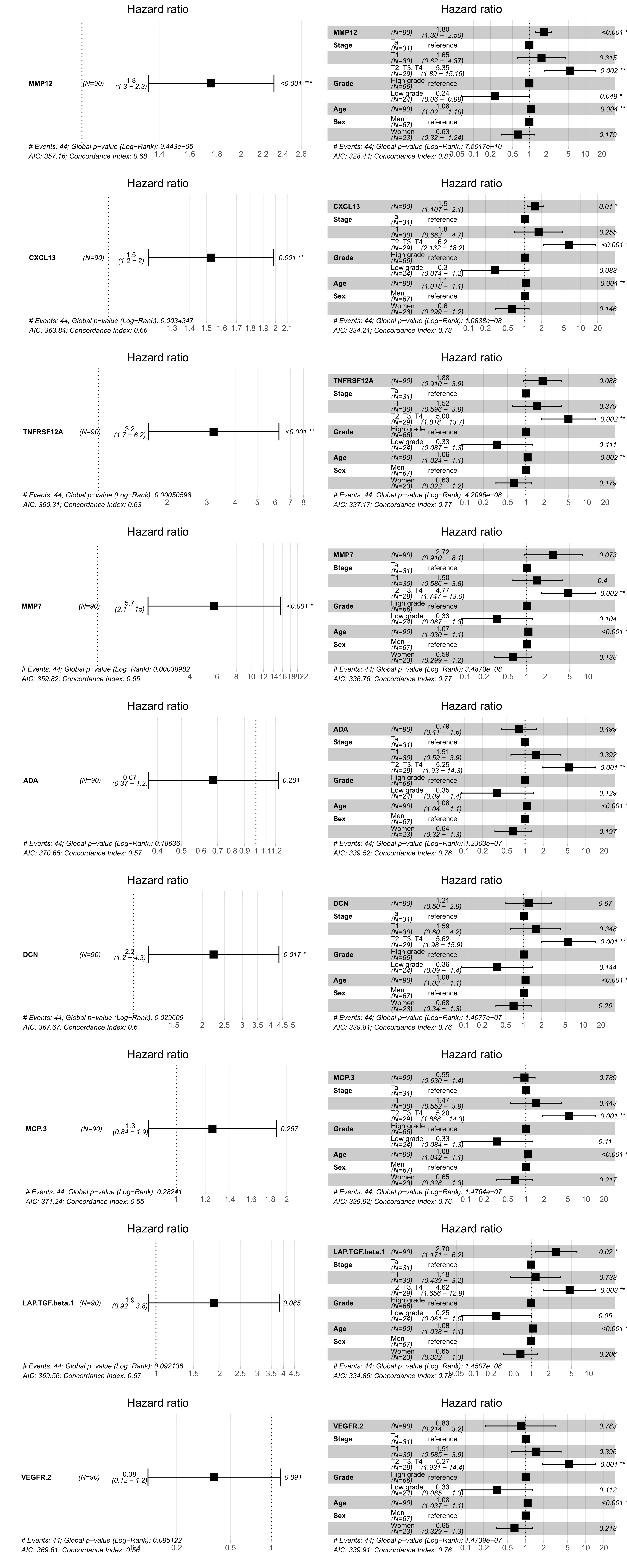

Supplement: Supplementary file 4 — Additional file 4: Supplementary Fig. 2. [file 12885_2023_11100_MOESM4_ESM.tiff]

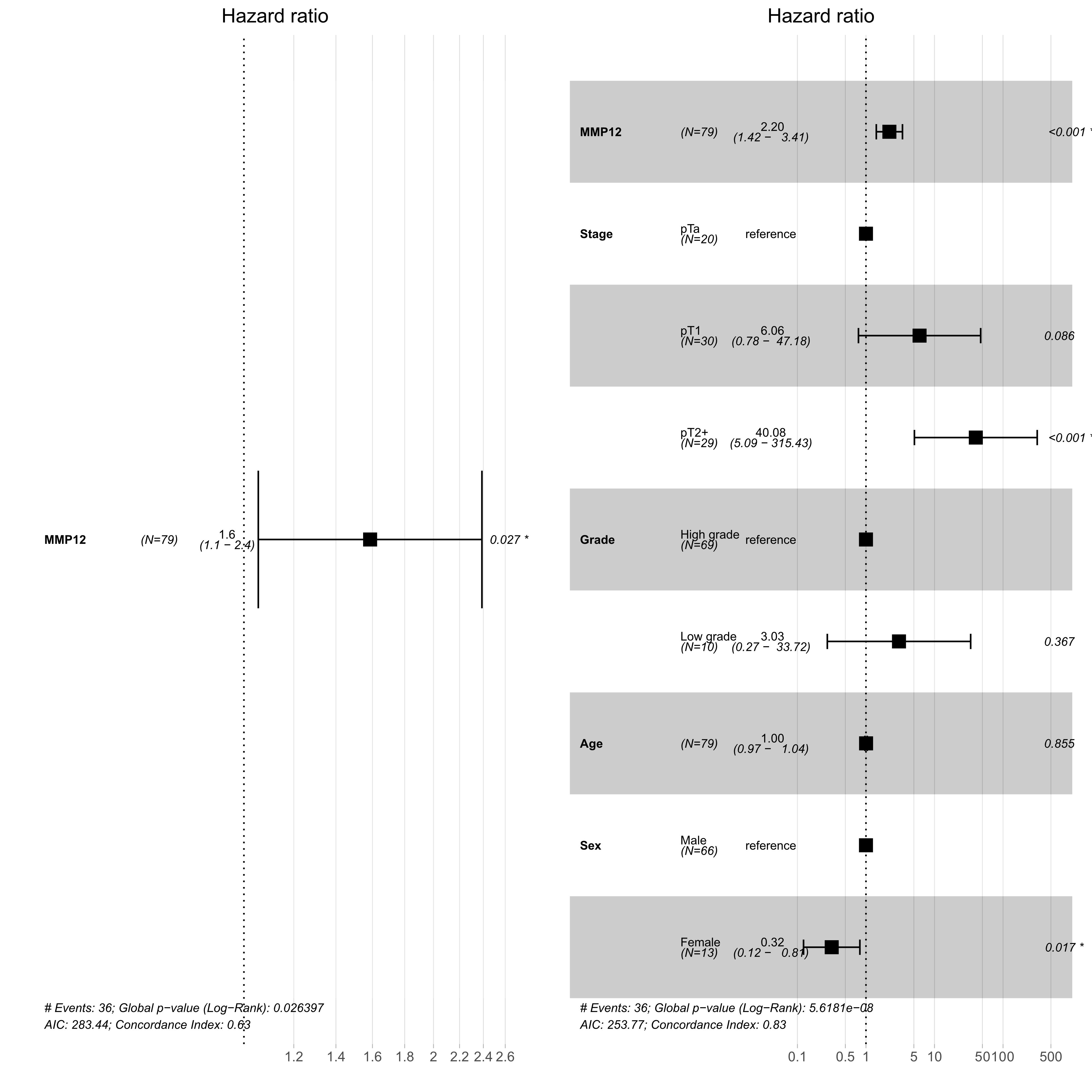

Supplement: Supplementary file 5 — Additional file 5: Supplementary Fig. 3. [file 12885_2023_11100_MOESM5_ESM.tiff]

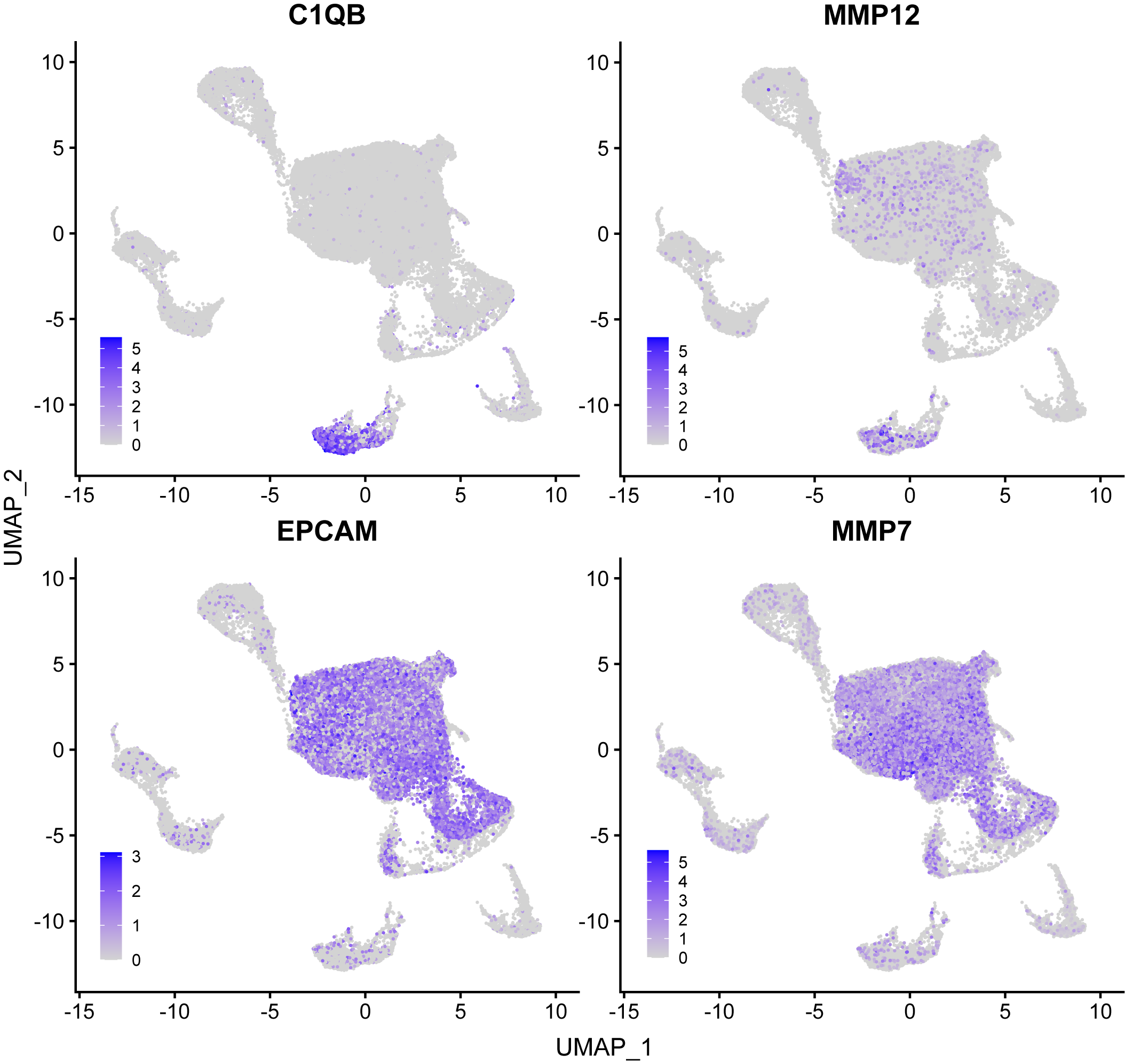

Supplement: Supplementary file 6 — Additional file 6: Supplementary Fig. 4. [file 12885_2023_11100_MOESM6_ESM.tif]

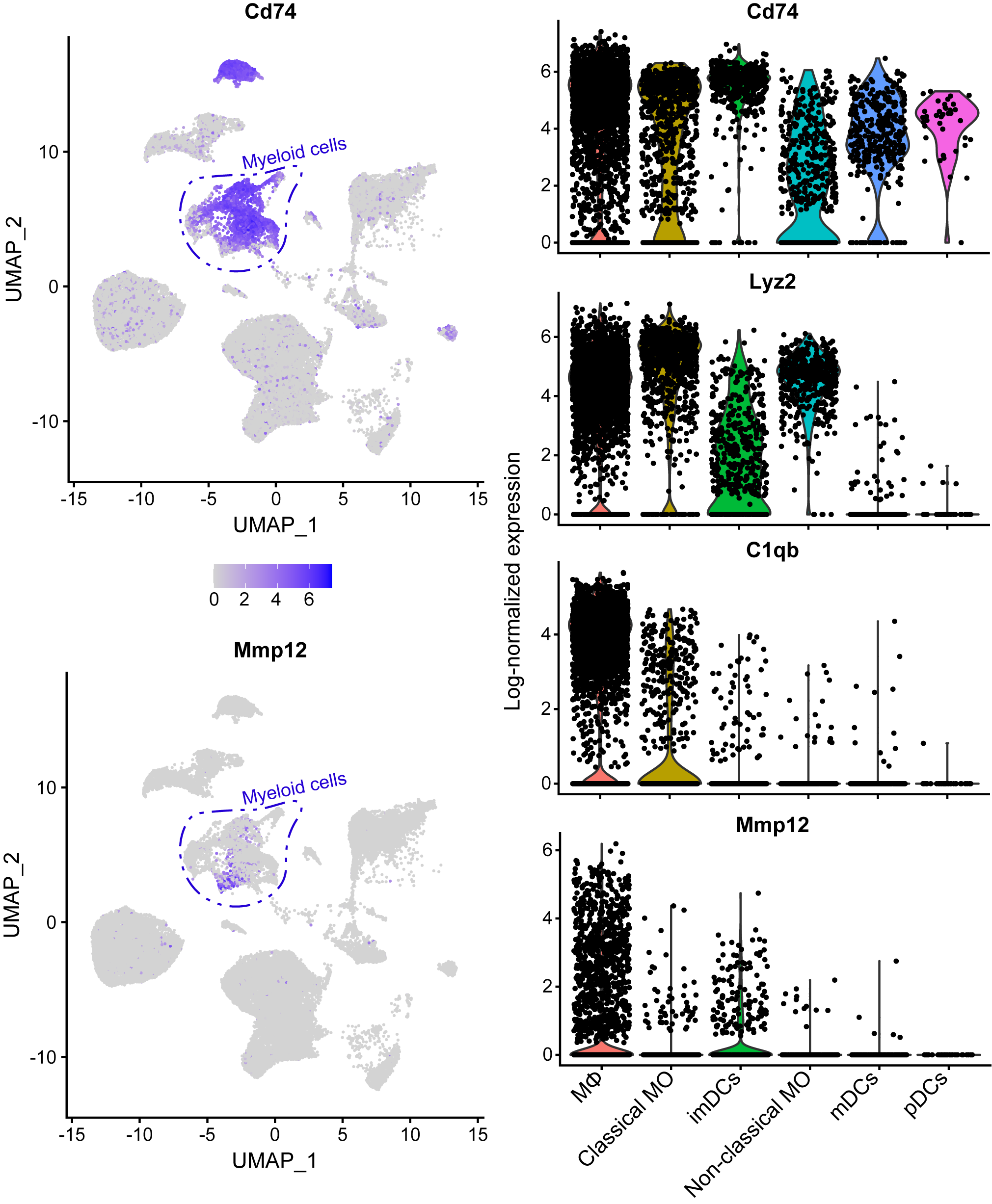

Supplement: Supplementary file 7 — Additional file 7: Supplementary Fig. 5. [file 12885_2023_11100_MOESM7_ESM.tif]
